# 

**Published:** 1912-12-07

**Authors:** 


### Buildings Contemplated.

Bradford.—Mr. F. Holland is architect for a children's home at Thackley for the Guardians. Tenders are invited and the contract contains a fair-wages clause.

Bromley.—The Bromley and Beckenham Joint Hospital Board has decided to provide a new isolation block.

Cheddleton.—The Staffs. County Council has approved an estimate of £7,191 for a nurses' home at Cheddleton Asylum.

Cornwall.—The County Council has approved an estimate of £10,500 for a consumptive sanatorium.

Dartmouth.—The Town Council has appointed a committee to confer with the Medical Officer of Health and the Borough Surveyor as to the provision and cost of an isolation hospital.

Hants.—The Local Government Board has sanctioned a loan of £39,348 for the foundation works of the joint asylum at Park Prewitt.

Hendon.—A tender of £2,074 is recommended for acceptance for the erection of a cottage hospital.

Lewes.—The East Sussex County Council's Health and Housings Committee has provisionally approved an estimate of £19,959 for a joint sanatorium of ninety-two beds.

Lincoln.—The Lincoln Board of Guardians has appointed a committee to negotiate for a site for a new infirmary.

London.—Alterations and extensions are proposed to Garratt Lane Workhouse boiler-house, etc., and twelve tenders have been considered by the Wandsworth Board of Guardians; the lowest is £2,492 10s., and the highest £3,657.

Rollesby.—The East and West Flegg Rural District Council is considering the establishment of a temporary isolation hospital for the Flegg Hundreds.

### Appointments.

THE HOSPITAL OF ST. JOHN OF JERUSALEM.

The King has been graciously pleased to sanction the following promotions in, and appointments to, the Order of the Hospital of St. John of Jerusalem in England:—As Chaplain: The Right Reverend the Bishop of Gibraltar, D.D. As Knights of Grace: Sir Ernest Frederick Schiff; Major Arthur William Mayo-Robson, C.V.O., F.R.C.S., R.A.M.C. (T.F.); William Henry St. John Hope, Esq. (from Esquire); Major-General Sir Geoffry Barton, K.C.V.O., C.B., C.M.G.; Frank Henry Cook, Esq., C.I.E. (from Esquire); Thomas Henry Woolston, Esq. (from Esquire). As Ladies of Grace: Lilian Margaret, Lady Clark; Agnes, Lady Jekyll; Constance Mary, Lady Knowles. As Esquires: William Disbrow Brydone-Jack, Esq., L.R.C.P., L.R.C.S.Ed. (from Honorary Associate); Major Dudley Henry Alexander, C.M.G.; Thomas Nelson, Esq., M.D. (from Honorary Associate); John Henry Rogers, Esq., A.C.A. (from Honorary Associate).

### Gifts and Bequests.

The Skinners' Company have sent £10 10s. to Queen Charlotte's Hospital.

The Queen, who is patron of the City of London Lying-in Hospital, has sent a parcel of linen for the benefit of the patients.

The workpeople of Messrs. N. Corah and Sons, hosiery manufacturers, of Leicester, have voted £400 to the funds of the Leicester Royal Infirmary.

The Chelsea Hospital for Women has received £105 from Amy, Lady Tate, and £25 each from Mr. and Mrs. Comyns Berkeley towards its Rebuilding Fund.

The Skinners' Company have given ten guineas to the After Care Association for poor persons discharged recovered from Asylums for the Insane, Church House, Westminster.

Mr. Thomas Crompton Waterhouse, dress goods merchant, has left £300 to the Northern Hospital, Manchester, "provided that such hospital is free of Governmental and municipal control at my death."

The Leicester Royal Infirmary has received £25 from Capt. the Hon. Sir W. C. Wentworth Fitzwilliam, K.C.V.O. (Equerry to the King), and £50 from Mrs. Lupton Topham to the Children's Hospital Reconstruction Fund.

Mr. Washington Epps, M.R.C.S., L.R.C.P., physician to the London Homœopathic Hospital and former President of the British Homœopathic Society, left estate valued at £36,948 gross, with net personalty £36,644.

### Obituary.

Surgeon-Colonel H. Martineau Greenhow has died at Esher in his eighty-fourth year. His father was for many years senior surgeon of the Newcastle-on-Tyne Infirmary, and the son took up the study of medicine, taking his M.R.C.S. in 1853. A Mutiny veteran, he formed one of the original garrison in the siege of Lucknow, and was strongly recommended for the V.C. He was one of four assistant surgeons, including the late Sir Joseph Fayrer, who were promoted to be brevet-surgeons "in consideration of their services during the siege of Lucknow." He became a Fellow of the Royal College of Surgeons of Edinburgh.

Lieutenant-Colonel Edward Alfred Birch, M.D., F.R.C.S., late of the Bengal Medical Service, has died in London. He joined the Indian Medical Service in 1866, and, after serving as civil surgeon, he was appointed, in 1885, surgeon-superintendent of the Presidency General Hospital, Calcutta. Some five years later he became Principal and Professor of Medicine at the Calcutta Medical College. He gained an extensive practice in Calcutta, and was the author of a popular treatise on "The Management of Children in India." He was appointed temporary Inspector-General of Hospitals in Bengal in November 1892, and he retired in 1893.

### Books Received.

Lynwood and Co., Ltd.: "Rag Time," by Lieut.-Col. J. D. F. Donegan.

Adlard and Son: "Mental Deficiency and the Mental Deficiency Bill, 1912."

University Tutorial Press: "Matriculation Directory," No. 62; "London University Press Calendar, 1913."

Methuen and Co., Ltd.: "Perfect Health for Women and Children," by Elizabeth S. Chesser; "The Malthusian Limit," by Edward Isaacson.

Eyre and Spottiswoode: "Winter's Pie, 1912."

S. W. Partridge and Co.: "First Steps to Nursing," by Mabel H. Cave.

James Maclehose and Sons: "A Manual of Immunity," by Elizabeth Fraser, M.D.

Hodder and Stoughton: "Another Device," by Stephen Paget.

Simpkin, Marshall, Hamilton, Kent and Co.: "Tested and Tried, or Cooking Made Easy," by Elsie Knight.

The Scientific Press, Ltd.: "Massage Manual," by Mabel M. Pireau.

University of London Press: "Health Resorts of the British Islands," by Neville Wood, M.D.

A. R. Mowbray and Co.: "In the Cardinal Ward," by A. Allen Brockington.

J. Wright and Sons (Bristol): "Pye's Surgical Handicraft," by Clayton Greene, 6th edition.

Society for the Propagation of the Gospel in Foreign Parts: "The Claim of the Suffering," by E. K. Paget.

W. H. and L. Collingridge: "The City Diary, 1913."

